# A Simplified Method for Anionic Surfactant Analysis in Water Using a New Solvent

**DOI:** 10.3390/toxics10040162

**Published:** 2022-03-29

**Authors:** Jung-Hwan Yoon, Yong-Geon Shin, Mary Beth Kirkham, Seok-Soon Jeong, Jong-Geon Lee, Hyuck-Soo Kim, Jae E. Yang

**Affiliations:** 1 Kangwon Institute of Inclusive Technology, Kangwon National University, Chuncheon 24341, Korea; yoonfnfg@hanmail.net; 2 Department of Biological Environment, Kangwon National University, Chuncheon 24341, Korea; jssddg888@naver.com (S.-S.J.); kimhs25@kangwon.ac.kr (H.-S.K.); 3 Industrial Wastewater Control Division, Gangwon Institute of Health and Environment, Chuncheon 24203, Korea; sindosa68@korea.kr (Y.-G.S.); solo3108@naver.com (J.-G.L.); 4 Department of Agronomy, Kansas State University, Manhattan, KS 66506, USA; kirkhammb@gmail.com

**Keywords:** anionic surfactant, analytical method, chloroform, interferences, MIBK-DCE (3:1)

## Abstract

Anionic surfactants (AS) are becoming a major emerging contaminant of waters due to their widespread use in household and industrial products. The standard chloroform method for analysis of AS in water relies on chloroform extraction of a methylene blue active substance (MBAS), which contains ion pairs between methylene blue (MB) molecules (positively charged) and AS. Due to the poor extractability of chloroform, the procedure is complicated, time-consuming, and subject to anionic interferences. A mixture of methyl isobutyl ketone (MIBK)–1,2-dichloroethane (DCE) at a 3:1 ratio of MIBK:DCE proved to be a robust solvent for AS extraction for a wide range of samples under various chemical conditions. The objectives of this research were to set the washing protocol to eliminate the anionic interferences in the MIBK-DCE extraction and to develop a new simplified analytical method for AS analysis using the MIBK-DCE (3:1) extractant. The suitability of the proposed MIBK-DCE method was validated based on quality control and assurance criteria, such as selectivity, accuracy, precision, method detection limit (MDL), limit of quantification (LOQ), and sensitivity. Various water samples, such as freshwater, wastewater, and seawater, were used for the method development and validation. Interferences by inorganic and organic anions were evident in the reference chloroform method but were eliminated in the MIBK-DCE procedure with a two-step process that consisted of washing with a carbonate/bicarbonate solution at pH 9.2 and a mixture of silver sulfate (Ag_2_SO_4_) and potassium alum (AlK(SO_4_)_2_). The simplified MIBK-DCE method for sodium dodecyl sulfate (SDS) analysis consisted of (i) sample pre-treatment, (ii) MIBK-DCE extraction, (iii) washing and filtration, and (iv) absorbance measurement. The MIBK-DCE method was accurate, precise, selective, and sensitive for AS analysis and showed MDL of 0.0001 mg/L, LOQ of 0.0005 mg/L, relative standard deviation (RSD) of 0.1%, and recovery of 99.0%. All these criteria were superior to those of the chloroform method. Sensitivity analysis showed highly significant correlations in AS analyses between the MIBK-DCE and chloroform methods for domestic wastewater, industrial wastewater, and seawater. The MIBK-DCE method is simple, rapid, robust, reproducible, and convenient, when compared to the chloroform method. Results demonstrate that the simplified MIBK-DCE method can be employed for AS analysis in a wide range of environmental waters including seawater.

## 1. Introduction

Anionic surfactants (AS), such as sodium dodecyl sulfate (SDS) and linear alkylbenzene sulfonates (LAS), are the most widely used surfactants compared to other types of surfactants, and they are found in personal care products, household detergents, and industrial products, such as carpet cleaners, because of their high detergency, foaming efficiency, and low cost [1,2,3,4]. They break surface tension and are the active washing component in detergents. Due to increasing use of detergents, AS are becoming a major, emerging contaminant in domestic wastewaters.

Aquatic environments are polluted with AS when domestic and industrial wastewaters are discharged into rivers [4,5,6]. The increased concentrations of AS in the environment is raising health concerns [1,7], because of the risk that they pose to biota and humans [4,8,9]. A rapid and accurate determination of AS in aquatic environments is required for the legislation and management of water quality [1,4].

The standard reference method for analysis of AS in water (chloroform method) is a spectrophotometric method that analyzes a methylene blue active substance (MBAS), which consists of ion-pairs formed between methylene blue (MB) and AS. In the method, a chloroform extractant is used [10,11,12,13,14]. However, this method has several drawbacks that are mainly due to the properties of chloroform and the presence of interferents [12,14,15].

Ideal criteria for the solvent to be used in the analysis of AS in different types of water are that it should have a high extractability, a short phase separation time, a low volatility, a low solubility in water, a lower density than water, and a lower potential health hazard [14,15,16]. However, chloroform has a low AS extraction efficiency and a relatively high density and volatility [10,14,15,16,17,18,19]. Yoon et al. [16] reported that no single solvent in SDS extraction could, among several solvents screened, meet such ideal criteria.

The chloroform method requires several back extractions with chloroform, because of its low extractability. This necessitates large volumes of water samples and chloroform and much glassware, all of which make the method complicated, labor-intensive, tedious, and time-consuming. In addition, this method is not suitable for multiple sample analyses [10,12,13,14,15,18].

Yoon et al. [16] showed that for a wide range of aquatic samples a mixture of two solvents, methyl isobutyl ketone (MIBK) and 1,2-dichloroethane (DCE), in a 3:1 ratio {MIBK-DCE (3:1)}, was selective and sensitive for SDS analysis, and it enhanced SDS extractability. As compared to chloroform, the solvent MIBK-DCE (3:1) had a lower volatility, a lower density than water, and a quicker phase separation. The solvent MIBK-DCE (3:1), when used in SDS extraction, was robust, because the extraction efficiency was independent of interference. It also was insensitive to changes in experimental conditions, such as pH and ionic strength. Thus, in place of chloroform, the MIBK-DCE (3:1) solvent could be used as an extractant in a new analytical method for AS.

Analyses of AS in water samples have, however, been challenging due to the complexity and variety of sample matrixes as well as surfactant properties [5]. In some cases, it is the sample’s matrix that determines the best method [20]. The chloroform method suffers from salt interference, for example chloride ions in sea water [12,14,21,22]. Yoon et al. [16] found that, for SDS extraction by MIBK-DCE (3:1), anionic interference from halogen ions and polyatomic and organic anions existed only at elevated concentrations, which do not occur in natural aquatic environments. A sample with a complex matrix, as found in wastewater and seawater, requires a method that has good selectivity and sensitivity for AS to avoid such anionic interferences [12,14,15,20].

The objectives of this paper were (1) to determine washing reagents that remove anionic interferences during AS extraction by MIBK-DCE (3:1), (2) to propose a new, simplified analytical method for AS in water using the MIBK-DCE extractant (MIBK-DCE method), and (3) to validate the selectivity and sensitivity of the MIBK-DCE method, when compared with the chloroform method, based on accuracy, precision, detection limit, recovery, and scale of operation for samples of freshwater, seawater, and domestic and industrial wastewaters.

## 2. Materials and Methods

### 2.1. Removal of Anionic Interferences

Elimination of anionic interferences by the washing process is the prerequisite step for analysis of AS in aquatic samples [10,11,12,13,14,16]. Any washing procedure should remove the methylene blue-anion (MB^+^A^−^) complex present in an aqueous or solvent phase. This study considered two possible options: (i) to remove anions (A^−^) in the water sample before the solvent extraction process, in order to inhibit the formation of MB^+^A^−^, either by charge modification of A^−^ with pH adjustment or by using a cation that has a high selectivity for A^−^ to form a (cation-A^−^) complex, and (ii) to remove the MB^+^A^−^ complex after solvent extraction by using a strong complexing reagent that forms a complex with A^−^ and destroys the MB^+^A^−^ ion pair.

Based on the above premises, four different stepwise washing approaches were taken to assess the washing efficiencies of various reagents on the removal of interferences from various anionic sources: (1) washing efficiency of deionized water, (2) washing efficiency of silver sulfate (Ag_2_SO_4_) when interferences were from halides, (3) washing efficiency using pH adjustment when interferences were from cyanide ion (CN^−^) and carboxylic organic acids, and (4) washing efficiency of a buffer solution and chelating agents when interferences were from phthalate and salicylate. These approaches were intended to eliminate all anionic interferences, in a stepwise fashion, from easily removable anions to anions that were difficult to remove. It is possible that they could co-exist in a complex matrix of environmental samples. Based on the washing efficiency of each reagent or process, the integrated washing reagents and procedures were determined. All chemicals used were reagent grade. All experiments were done at room temperature and in triplicate unless otherwise stated.

#### 2.1.1. Washing Efficiency of Deionized Water on Interferences from Anions

Solutions of sodium fluoride (NaF), sodium chloride (NaCl), potassium bromide (KBr), potassium iodide (KI), sodium nitrite (NaNO_2_), potassium nitrate (KNO_3_), potassium cyanide (KCN), potassium dihydrogen phosphate (KH_2_PO_4_), sodium bicarbonate (NaHCO_3_), sodium acetate (CH_3_COONa), potassium sodium tartrate (KNaC_4_H_4_O_6_·4H_2_O), trisodium citrate (Na_3_C_6_H_5_O_7_·2H_2_O), sodium benzoate (C_6_H_5_COONa), potassium biphthalate (C_6_H_4_COOHCOOK), or sodium salicylate (C_6_H_4_OHCOONa) were prepared (Table 1 and [16]). All solvents were purchased from Wako Pure Chemicals (Tokyo, Japan). Concentrations (M) of anions higher than those present in natural water were used in this study to ensure that there were anionic interferences (see Table 1). Inorganic anions generally exist in natural water in the range of μM–mM [23,24,25], and concentrations of organic anions are present in the nM–μM range [25,26,27,28,29].

One hundred mL of each solution was transferred into two different separatory funnels (Batch-I and Batch-II), and, thereafter, the pH of each solution was adjusted to neutral using dilute H_2_SO_4_ or NaOH. To each batch, 5 mL of 0.025% methylene blue (MB: C_16_H_18_N_3_SCl·3H_2_O, Wako Pure Chemicals, Tokyo, Japan) was added to allow for the formation of the MB and anion complex (MB^+^A^−^). Thereafter, 50 mL of MIBK-DCE (3:1) extractant was added and each batch was shaken for one minute. After a complete phase separation, the aqueous layer was discarded. The MIBK-DCE layer was washed once with 50 mL of deionized water for Batch-I, followed by shaking for 1 min. Settling was allowed until there was a complete phase separation, and then the aqueous layer was discarded. For Batch-II, no washing was done. The extracted MIBK-DCE layer was filtered through Whatman® 1PS water repellent phase separating filter paper (Sigma-Aldrich, St. Louis, MO, USA). Absorbances of the filtrate were measured at 658 nm using a spectrophotometer (DU800, Beckman Coulter, Indianapolis, IN, USA). A standard curve was separately constructed between sodium dodecyl sulfate (SDS) concentrations and absorbances. Absorbances of the filtrate were interpolated into the SDS standard curve to calculate the [MB^+^A^−^] concentrations, which were equivalent to the interfering concentration of each anion. The MB and MIBK-DCE solutions were in brown-colored bottles and were always kept in a refrigerator.

#### 2.1.2. Washing Efficiency of Silver Sulfate (Ag_2_SO_4_) on Interferences from Halogen-Ion

Solutions of 0.5 M NaF, 1.0 M NaCl, 0.5 M KBr, or 0.001 M KI were prepared, and 100 mL of each solution was transferred to separatory funnels allowing for the formation of MB^+^A^−^ complexes by using the same procedure as described in (Section 2.1.1). The MIBK-DCE layer was washed once with 50 mL silver sulfate (Ag_2_SO_4_, Wako Pure Chemicals, Tokyo, Japan) and filtered through Whatman^®^ 1PS filter paper. Absorbance of each filtrate was measured and interpolated into the SDS standard curve to calculate the [MB^+^A^−^] concentrations. Interfering concentration of each halogen ion could be determined.

#### 2.1.3. Washing Efficiency of pH Adjustment on Interferences from Cyanide ion (CN^−^) and Carboxylic Organic Acids

Chemical species of CN^−^ and organic acids are subject to changes by pH and thus affect the formation of MB^+^A^−^ complexes [30,31,32,33]. To assess the pH effects on removal of CN^−^ interferences, a 0.1 M KCN solution was prepared, and the pH was adjusted to 1.2–10.3 using dilute H_2_SO_4_ or NaOH. The extraction procedures for the MB^+^A^−^ ion pairs were the same as those described in (Section 2.1.1). The MIBK-DCE solvent layer was washed once with deionized water.

For the removal of interferences from organic anions, such as acetate, tartrate, citrate, benzoate, salicylate, or phthalate, each solution was prepared, and each solution was adjusted to about pH 3 by adding the pre-treatment reagent. The pre-treatment reagent was prepared by mixing 50 mL of 0.5 M H_2_SO_4_ and 49.3 g MgSO_4_·7H_2_O (0.2 mole) and diluting to 1 L with deionized water to acidify the sample and maintain a constant ionic strength of the solution [16]. The extraction procedure for the MB^+^A^−^ ion pairs was the same as described in (Section 2.1.1).

Absorbances measured for MB^+^A^−^ in the solvent layer were interpolated into the SDS standard curve to calculate the SDS concentrations that were equivalent to the interfering concentrations of CN^−^ and organic acids.

#### 2.1.4. Washing Efficiency of Buffer Solution and Complexing Agents on Interferences from Phthalate and Salicylate

A carbonate–bicarbonate buffer solution (pH 9.2) was prepared by mixing 4 mL of 0.2 M Na_2_CO_3_ and 46 mL of 0.2 M NaHCO_3_ solutions. Ten kinds of buffer solutions were screened, for example, phosphate buffer, boric acid–borax buffer, borax–NaOH buffer, etc., but the carbonate–bicarbonate buffer solution was selected due to its better washing efficiency compared to the others (data not shown).

To inhibit interferences by phthalate and salicylate by allowing them to form a cation-A^−^ complex, 0.05 M of AlK(SO_4_)_2_, MnSO_4_, and ZnSO_4_ solutions were compared, in which Al, Mn, and Zn are known to form a stable complex with salicylate or phthalate [23,25,34]. Extractions of MB^+^A^−^ and quantification of interferences by MB^+^A^−^ were the same as before (Section 2.1.1).

#### 2.1.5. Development of Washing Agents

Based on the washing efficiencies of the above washing reagents on removal of interferences of MB^+^A^−^, an optimal combination of the washing reagents for the MIBK-DCE analytical method was recommended.

### 2.2. Development of MIBK-DCE Method for the Analysis of AS

Based on the extraction efficiencies of the MIBK-DCE extractant under different experimental conditions [16] and the removal efficiencies of different washing processes for the anionic interferences (this study), a new MIBK-DCE analytical method for AS was proposed. The new MIBK-DCE method is a modified and simplified procedure compared to the standard reference method [10,11,13].

**Figure 1:**
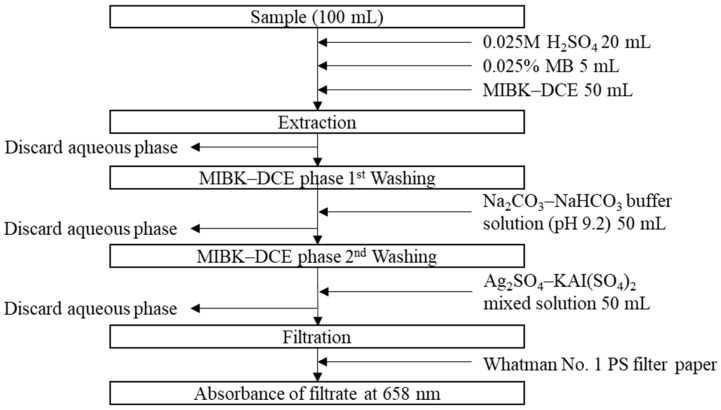
Experimental procedures of the MIBK-DCE method.

Figure 1 shows the flowchart of the MIBK-DCE method. This method consists of the following steps: (i) sample pre-treatment, (ii) extraction, (iii) washing and filtration, and (iv) absorbance measurement. In this method, one separatory funnel per sample is necessary. No back-washing process is needed.

A sample of 100 mL was put into separatory funnel, followed by adding, in series, 20 mL pre-treatment reagent (pH 3), 5 mL 0.025% MB, and 50 mL MIBK-DCE (3:1) solvent. The mixture was shaken for 1 min and allowed to settle for a few minutes until the aqueous and solvent layers were clearly separated. After discarding the aqueous phase layer, 50 mL of the first washing reagent (Na_2_CO_3_–NaHCO_3_ buffer solution at pH 9.2) was added to the MIBK-DCE layer, followed by shaking for 1 min and phase separation. After discarding the aqueous phase, the second washing process using 50 mL of the Ag_2_SO_4_ and AlK(SO_4_)_2_ mixture was repeated as the first washing process. The aqueous layer was then discarded. The extracted MIBK-DCE layer was filtered through a water-repellent, phase-separating paper (Whatman^®^ 1PS, Sigma-Aldrich, St. Louis, MO, USA). Absorbances of the filtrate were measured at 658 nm using a spectrophotometer (DU800, Beckman Coulter, Indianapolis, IN, USA). Results from the MIBK-DCE method were compared with those from the chloroform method [10,11,13].

### 2.3. Suitability of the MIBK-DCE Method for the Analysis of AS

#### 2.3.1. Validation of the MIBK-DCE Method

Results of the MIBK-DCE method were validated based on quality control (QC) and quality assurance (QA) protocols recommended by Korea MOE [13], APHA [10], Wisconsin Department of Natural Resources [35], Rao [36], and Swartz and Krull [37]. These QC/QA criteria include accuracy, precision, recovery, method detection limit (MDL), and limit of quantification (LOQ). In addition, scale of operation, such as analysis time and amounts of solvent and glassware needed, which are required for analyses of every twelve sample analyses, were assessed for both the MIBK-DCE and chloroform methods. The twelve samples consisted of six SDS standard solutions and six natural-water samples.

#### 2.3.2. Sensitivity Analysis of the MIBK-DCE Method

Sensitivity of the MIBK-DCE method was verified from the correlation between SDS concentrations measured by the MIBK-DCE method and the chloroform method for various water samples. For this, 24 domestic sewage water samples and 17 industrial wastewaters from a car wash and laundry were taken from several locations in Gangwon Province, Korea (Table S1). Seawater samples were taken from the East Sea (Gangreung City) of Korea. Because the concentration of AS in seawater samples was lower than the MDL (0.02 mg/L) for the chloroform method, standard solutions of SDS were spiked into the natural seawater sample matrix and the SDS concentrations were determined by the two methods. Sensitivity was evaluated by the goodness-of-fit (significantly higher coefficient of determination) and a low standard error.

## 3. Results and Discussion

### 3.1. Removal Efficiency of Anionic Interferences

Various inorganic and organic anions (A^−^) that co-exist in aqueous samples form MB^+^A^−^ complexes. If these complexes are present in the MIBK-DCE solvent phase, they interfere in the analytical results and result in a higher AS analysis than expected [10,12,13,14]. Cations in water (C^+^_(aq)_) form C^+^AS^−^ ion pairs with anionic surfactants (AS^−^_(aq)_), but their interfering effect on AS extraction by MIBK-DCE was negligible as compared to that by anions [16]. Any analytical method should remove MB^+^A^−^ ion pairs to reduce error [12,14,15]. The reference chloroform method requires several back-washing processes with deionized water to remove such interferences, but the washing efficiency was, in many cases, unsatisfactory [10,12,13,14,15]. This might be related to the insufficient partitioning capacity of water among A^−^, MB^+^A^−^, and MB^+^AS^−^.

#### 3.1.1. Washing Efficiency of Deionized Water on Interferences from Anions

Table 1 shows the effect of washing the MIBK-DCE layer by deionized water on the interference removals caused by 15 anions. Anion concentrations treated were much higher than those present in the natural aquatic water samples [16]. Deionized water lowered MB^+^A^−^ concentration substantially in the solvent layer, as compared to no-washing. When deionized water was added to the MIBK-DCE layer that contained the MB^+^A^−^ complex, MB^+^A^−^ redistributed to either the aqueous phase or the solvent phase, depending upon the partitioning coefficients. When the aqueous layer was discarded, the MB^+^A^−^ partitioned into the aqueous layer was removed, resulting in a lowering of the interferences. In case of F^−^, washing reduced the interference from 0.05 to 0.01 mg/L, which was lower than the detection limit of the chloroform method (0.02 mg/L).

For the rest of the anions tested, washing the solvent layer once with deionized water was not sufficient to remove the interferences below the method detection limit. Results indicate that an alternative way for further washing processes is needed to eliminate the residual anionic interferences.

#### 3.1.2. Washing Efficiency of Silver Sulfate (Ag_2_SO_4_) for Halide Interferences

For the residual anionic interferences that could not be lowered by deionized water, many kinds of Ag^+^ solutions, for example AgNO_3_ and Ag_2_SO_4_, were screened to select a better reagent to destroy the MB^+^A^−^ complex and convert it into Ag^+^A^−^ to reduce the interferences (Equation (1)).

MB^+^A^−^_(ion-pair)_ + Ag^+^_(aq)_ → AgA_(s)_↓ + MB^+^_(aq)_ (1)

The silver ion has a high stability constant, especially with halogen ions (A^−^) such as F, Cl, Br, and I [23,25,33,38]. Among reagents screened, Ag_2_SO_4_ showed the best performance in breaking the MB^+^A^−^ complex and forming an Ag^+^A^−^ complex (data not shown). Table 2 shows that washing the MIBK-DCE layer with 0.001 M Ag_2_SO_4_ almost eliminated the halogen ion interferences (compare results in Table 1), and they were lower than the method detection limit (0.02 mg/L) of the chloroform method, even though halide concentrations were much higher than those in natural water samples. The Ag^+^A^−^ complex from the MB^+^A^−^ ion pair was partitioned into the aqueous solution and then discarded. This was due to the high stability constants (low solubility product constants) of Ag with halogen ions.

However, it was necessary to confirm that 0.001 M Ag_2_SO_4_ as a washing reagent could transform only the MB^+^A^−^ (halogen) ion pair (Equation (1)) but not the MBAS ion pair. Results showed that the SDS standard curve maintained the highly significant linearity {y = 0.7422x − 0.002; r^2^ = 0.9999 ** (*p* < 0.001)} even when 0.001 M Ag_2_SO_4_ was used as a washing reagent. This shows that the Ag ion in Ag_2_SO_4_ selectively deprived the halides from the MB^+^A^−^ ion pairs without affecting the MBAS complex.

#### 3.1.3. Washing Efficiency of pH Adjustment on Interferences from Cyanide ion (CN^−^) and Carboxylic Organic Acids

The residual interferences by CN^−^ and polyprotic organic acids after washing the MIBK-DCE layer with deionized water were remarkable (Table 1). To avoid CN^−^ interference, a method to inhibit the formation of the MB^+^-CN^−^ ion pair is needed when MB is added, and this was tested by changing the charge of CN^−^ in advance with pH adjustments.

The speciation of cyanide is subject to pH [30,31,32,33]. Free cyanide refers to the sum of hydrogen cyanide (HCN) and cyanide ion (CN^−^) in a sample. At pH 7 or less in water, free cyanide is present entirely as HCN. Above pH 11, free cyanide exists entirely as CN^−^ [39,40]. HCN does not have an ionic charge and, thus, cannot form the complex with MB, potentially showing no interference in AS analyses.

Table 3 shows the pH effects on the CN^−^ interference in the analysis of AS by the MIBK-DCE method. When the pH of water samples was in the acidic range, the interferences of CN^−^ were lower than those under alkaline conditions. This might be due to a lower availability of CN^−^ species in acidic conditions. For residual CN^−^ inhibition that was not removed by acid treatment, washing with the Ag_2_SO_4_ solution was enough to lower the interference, as shown in Table 4.

For those polyprotic anions, it was necessary to see if the pH adjustment might enhance or inhibit their interferences because pH may change the charges of these anions. Table 5 shows the effect of the pre-treatment reagent on interferences by acetate, tartrate, citrate, benzoate, salicylate, and biphthalate. Adjustment of pH eliminated interferences by aliphatic carboxylic anions, such as acetate, tartrate, and citrate. However, pH adjustment could not effectively eliminate interferences by the aromatic carboxylic anions, such as benzoate, salicylate, and biphthalate. The interferences by salicylate and biphthalate were still prominent. At pH 3, total phthalate exists as a mono-valent anion species (C_6_H_4_COOHCOO^−^) that can form an MB-phthalate complex [32].

In the chloroform method, the MOE method [13] modified the methods of APHA [10] and EC [11] by including pre-treatment steps that are excluded from the APHA and EC procedures. The pre-treatment steps prepare the mixture of alkaline boric acid (500 mL of 0.05 M Na_2_B_4_O_7_·10H_2_O + 500 mL of 0.4% NaOH) solution, 0.025% MB, and chloroform before sample extraction by chloroform. The pre-treatment steps are taken to remove the interferences that might occur from transformation of MB under alkaline conditions. The chloroform method recommends extracting MBAS by chloroform under alkaline condition [10,11,13]. However, when MB coexists with chloroform at a pH higher than 9.5, MB is transformed into dimethylthionoline, which shows a reddish color and, thus, interferes with the quantification of MBAS [41,42]. When the reddish color is evident, additional washing steps with chloroform are repeatedly required until a clear color is shown. This step causes a longer analytical time, even though it may remove the interference from the transformed MB. In the MIBK-DCE method, the pre-treatment reagent under acidic conditions assures that no such interference will occur [16].

#### 3.1.4. Effects of Buffer Solution and Complexing Reagents on Salicylate and Biphthalate Interferences

In natural waters or industrial wastewater, various kinds of organic acids are present and react with cations to form complexes [25,26,27,28,29]. As shown in Table 5, interferences by phthalate and salicylate could not be controlled by pH adjustment, possibly due to metal–ligand interaction. When a carbonate–bicarbonate buffer solution at pH 9.2 was used as the first washing reagent and was added to the MIBK-DCE phase, the 0.005 M phthalate interferences were decreased from 1.013 mg/L to 0.030 (Table 6).

This result might be related to the chemical species of the phthalate ion at different pHs. Under acidic conditions at around pH 3–5, species of phthalate exist as monovalent anions (C_6_H_4_COOHCOO^−^) that form an MB-phthalate ion pair (Equation (2)), depending upon pK_a_ values (Table 5). This ion pair partitions into the MIBK-DCE phase causing an interference. However, when the pH is higher than 6, phthalate species become a divalent anion {C_6_H_4_(COO)_2_^2−^}. Thus, when a carbonate–bicarbonate buffer solution (pH 9.2) was added, the neutral form of MB^+^ C_6_H_4_COOHCOO^−^_(ion pair)_ was transformed into MB^+^ C_6_H_4_(COO) _2_^2−^_(aq)_ (Equation (3)) that partitioned into the aqueous layer that was discarded. This might be a cause for reduction of phthalate inhibition by alkaline buffer solutions.

MB^+^_(aq)_ + C_6_H_4_COOHCOO^−^_(aq)_ ↔ MB^+^ C_6_H_4_COOHCOO^−^_(ion pair)_ (2)

MB^+^ C_6_H_4_COOHCOO^−^_(ion pair)_ + OH^−^ ↔ MB^+^ C_6_H_4_(COO)_2_^2−^_(aq)_ + H_2_O (3)

However, the residual interference by salicylate still existed (Table 6), possibly due to a high stability constant of salicylate with cations [25,27,30,32,34]. In this case, it was speculated that a cation, which has a stronger affinity for salicylate than MB, might destroy the MBAS-salicylate complex to remove the interference. Salicylic acid has two ionizable functional groups, i.e., OH and COOH. Depending upon pH and pK_a_ values, salicylate as a ligand (HL) forms three types of complexes with a metal (M): (i) M(HL)-complexes under a strong acidic condition, (ii) ML-chelates under a weak acidic condition, and (iii) M(OH)L complexes under an alkaline condition. Additionally, salicylate is known to form a strong complex with metal ions, such as with Fe^3+^, Cu^2+^, Be^2+^, and Al^3+^ [25,27,34].

As shown in Table 7, the interference by salicylate (0.01 M) was suppressed from 0.294 mg/L to 0.113 mg/L with the combined use of the carbonate–bicarbonate buffer solution as the first washing agent, and the complexing reagent [0.1% Ag_2_SO_4_ + 0.005 M AlK(SO_4_)_2_] as the second washing reagent. Salicylate is known to form a stronger complex with Al than with Mn and Zn [25,34]. Washing reagents containing Mn and Zn showed no effect on salicylate interferences.

#### 3.1.5. Development of Washing Reagents

Based on the washing efficiencies of various reagents and chemical conditions on the removal of interferences from inorganic and organic anions, we integrated the washing processes into three categories. In this study, we tested the anionic interferences at highly elevated concentrations, which hardly occur in natural water environments. In the following section, total reduction of anionic interferences by three processes was confirmed through validation experiments using the MIBK-DCE method and using SDS standard solutions and natural water samples.

(i) Pre-treatment reagent: 0.025 M H_2_SO_4_ + 0.2 M MgSO_4_: This reagent is used to treat the sample before the extraction step. It is intended for reduction of interferences by CN and organic acids.
(ii) First washing reagent: 20 mL of 0.2 M Na_2_CO_3_ was mixed with 230 mL of 0.2 M NaHCO_3_ and leveled to 1000 mL. This buffer solution is for maintaining the pH of the solution at 9.2 and preventing interferences from biphthalate and multi-elemental anions such as nitrate ion.
(iii) Second washing reagent: 1.0 g silver sulfate (Ag_2_SO_4_) and 2.0 g potassium alum (AlK(SO_4_)_2_) were dissolved in 1000 mL of hot water and cooled at room temperature, followed by adding 1 mL of 0.05 M H_2_SO_4_. Use of Ag_2_SO_4_ and AlK(SO_4_)_2_ prevent interferences from halogen ions, the CN ion, and the salicylate ion.

### 3.2. Validation of the MIBK-DCE Method

#### 3.2.1. QC/QA Criteria of MIBK-DCE Method

To validate the new MIBK-DEC method (Figure 1), QC/QA criteria of the analysis, such as method detection limit (MDL), limit of quantification (LOQ), accuracy, and precision, were determined (Table 8). Extraction efficiency of MIBK-DCE was better than that of chloroform as evidenced by a higher slope and coefficient of determination (r^2^) of the SDS standard curve.

The MIBK-DCE method revealed that MDL and LOQ were 0.0001 mg/L and 0.0005 mg/L, respectively. Precision, represented as the percentage of the relative standard deviation (RSD, %), and accuracy, represented as the percentage of recovery, were 0.1% and 99.0%, respectively. All these criteria were superior to those of the chloroform method, even though both methods satisfied the QC/QA targets of the chloroform method [10,13] (APHA, 2005; MOE, 2017), which are LOQ of 0.02 mg/L, precision of ±25%, and accuracy of 75–125%. Results demonstrate that the MIBK-DCE method is selective, accurate, and precise.

#### 3.2.2. Recovery of MIBK-DCE Method

Table 9 reveals the SDS recovery (%) using the MIBK-DCE method for various environmental samples, such as groundwater, stream water, wastewaters, and seawater that were taken from Gangwon Province, Korea. For assuring a reasonable range of analysis, natural water samples were spiked with specified SDS concentrations before analysis. The SDS concentrations in most samples, without spiking, were trace, except for wastewater. The recovery percentages were in the range of 98–103% (average 99%) with a low standard deviation. Results demonstrated that the MIBK method is applicable for the analysis of AS in a wide variety of environmental water samples, especially seawater that has a more complicated matrix than freshwater.

#### 3.2.3. Scale of Operation of MIBK-DCE Method

Table 10 shows the results on the scale of operation between the MIBK-DCE method and the chloroform method, such as analysis time, volume of solvent necessary, and glassware needed for twelve samples. The MIBK-DCE method reduced the analytical time from 270 min to 90 min, the solvent volume from 1390 to 600 mL, and the number of separatory funnels from 36 to 12. The MIBK-DCE method needs one separatory funnel per sample, but the chloroform method needs two separatory funnels and one extra piece of laboratory ware for sample treatment per sample. In addition to these criteria, the SDS extraction efficiency by the MIBK-DCE method was superior to that of the chloroform method [16].

#### 3.2.4. Sensitivity Analysis

**Figure 2:**
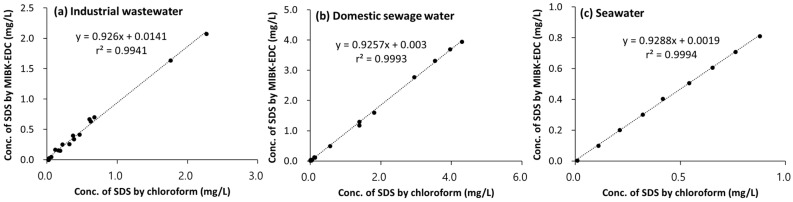
Sensitivity analysis between MIBK-DCE and chloroform method for the anionic surfactants in (**a**) industrial wastewater (*n* = 17), (**b**) domestic sewage water (*n* = 24), and (**c**) seawater (*n* = 9).

Figure 2 shows the sensitivity analysis in which SDS concentrations analyzed by the MIBK-DCE method and chloroform method were correlated for domestic sewage water, industrial wastewater (car wash), and seawater. There existed a highly significant correlation between the two methods. Slopes were slightly lower than 1 with a highly significant r^2^. The SDS concentrations determined by the chloroform method were slightly higher than those determined by the MIBK-DCE method. Results indicated that the two methods had a similar sensitivity for SDS analysis.

**Figure 3:**
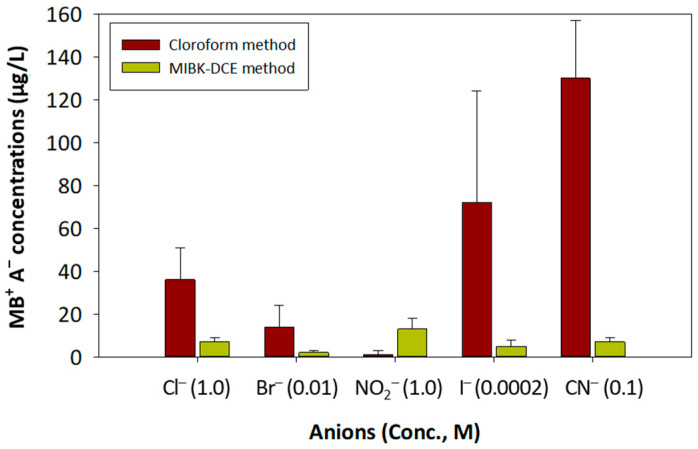
Comparison of the interfering concentrations of several inorganic anions that were measured by MIBK-DCE and chloroform methods.

To compare the robustness of the two methods, Figure 3 compares the interfering concentrations of several inorganic anions that were measured by the MIBK-DCE and chloroform methods. The MIBK-DCE method had lower interfering concentrations than the chloroform method for the anions tested, except for nitrite. The nitrite interferences by the two methods were, however, lower than the MDL (0.02 mg/L) of the chloroform method. All MB^+^A^−^ concentrations analyzed by the MIBK-DCE method were lower than the MDL of the chloroform method. Results indicated that the MIBK-DCE method was more robust than the chloroform method, because the MIBK-DCE method was less subject to anionic interferences than the chloroform method. Also, results demonstrated that the new method can be applicable to analysis of AS in seawater and industrial wastewaters that contain a high level of the Cl ion.

## 4. Conclusions

A simple, rapid, accurate, cost-effective, and precise analytical method for AS in water using MIBK-DCE solvent (MIBK-DCE method) was developed and validated for its sensitivity and selectivity using freshwater, seawater, and wastewater. An acidic pre-treatment reagent and two types of washing reagents effectively reduced all interferences of inorganic and organic anions below the MDL of the reference chloroform method. The MIBK-DCE solvent could replace the use of chloroform in analysis of AS by not only enhancing the SDS extractability but also by improving the physicochemical properties that provide favorable conditions for the analytical operation. The MIBK-DCE method is robust and reproducible and consists of four steps: sample pretreatment, extraction, washing and filtration, and absorbance measurement. The MIBK-DCE method eliminated a back-washing process, reduced the excessive use of solvents and laboratory glassware, and shortened the analytical time. The MDL, LOQ, relative standard deviation (RSD: %), and recovery (%) of the MIBK-DCE method were 0.0001 mg/L, 0.0005 mg/L, 0.1%, and 99%, respectively. All these criteria were superior to those of the chloroform method. There existed a highly significant correlation for SDS concentrations analyzed by the MIBK-DCE method and the chloroform method for domestic sewage water, industrial wastewaters, and seawater. Results demonstrated that the MIBK-DCE method permits accurate and rapid analysis of AS, without anionic interferences, in a wide variety of natural water samples. It has high selectivity, sensitivity, and reproducibility and can be carried out in a simple and less time-consuming way when compared with the chloroform method.

## Figures and Tables

**Table 1:** Washing efficiency of deionized water on the anionic interferences in MIBK-DCE extraction.

Anions and Treatment Concentrations (M)		Without Washing	Washing Once
		Interfering MB^+^A^−^ Concentration ^†^ (mg/L)	Interfering MB^+^A^−^ Concentration ^†^ (mg/L)
F^−^	0.5	0.049 ± 0.002	0.013 ± 0.001
Cl^−^	1.0	0.972 ± 0.015	0.065 ± 0.005
Br^−^	0.02	0.722 ± 0.012	0.101 ± 0.002
I^−^	0.0002	1.250 ± 0.010	0.206 ± 0.006
NO_2_^−^	0.25	0.932 ± 0.011	0.113 ± 0.006
NO_3_^−^	0.0025	0.825 ± 0.007	0.048 ± 0.005
CN^−^	0.2	0.647 ± 0.046	0.366 ± 0.052
H_2_PO_4_^−^	1.0	0.174 ± 0.004	0.086 ± 0.005
HCO_3_^−^	1.0	0.143 ± 0.003	0.058 ± 0.005
Acetate	1.0	0.203 ± 0.023	0.029 ± 0.001
Tartrate	0.5	0.162 ± 0.020	0.068 ± 0.015
Citrate	0.5	0.262 ± 0.036	0.102 ± 0.007
Benzoate	0.05	0.524 ± 0.018	0.073 ± 0.014
Biphthalate	0.05	0.899 ± 0.123	0.180 ± 0.015
Salicylate	0.0002	1.082 ± 0.016	0.253 ± 0.001

^†^ MB^+^A^−^ concentration, that is equivalent to interfering concentration of anions, was calculated by determining the absorbance of each MB^+^A^−^ solution and then interpolating the values into a SDS standard curve.

**Table 2:** Effect of washing with Ag_2_SO_4_ on reduction of interference by halides in MIBK-DCE extraction *.

Anions and Treatment Concentrations (M)		Interfering MB^+^A^−^ Concentrations ^†^ (mg/L)
F^−^	0.5	0.002 ± 0.001
Cl^−^	1.0	0.007 ± 0.002
Br^−^	0.5	0.016 ± 0.002
I^−^	0.001	0.011 ± 0.001

^†^ MB^+^A^−^ concentration, which is equivalent to interfering concentration of anions, was calculated by determining the absorbance of each MB^+^A^−^ solution and interpolating it into the SDS standard curve. * See Table 1 for the interfering MB^+^A^−^ concentrations without washing.

**Table 3:** Effect of pH on cyanide ion (CN^−^) interferences in MIBK-DCE extraction **^†^**.

pH	1.2	1.4	1.7	2.7	5.9	8.6	9.2	10.3
MB^+^CN^−^ * (mg/L)	0.098 ± 0.024	0.130 ± 0.091	0.084 ± 0.008	0.082 ± 0.024	0.130 ± 0.005	0.715 ± 0.133	0.564 ± 0.006	0.314 ± 0.113

^†^ The treated concentration of CN was 0.10 M and pH adjustment was made by H_2_SO_4_ or NaOH solutions. * MB^+^CN^−^ concentration, equivalent to the interfering concentration of CN^−^, was calculated by using the absorbance of each MB^+^CN^−^ solution and interpolating the values into an SDS standard curve.

**Table 4:** Effect of Ag_2_SO_4_ washing on cyanide ion (CN^−^) interferences in MIBK-DCE extraction.

Anions	Washing Reagents		Interfering MB^+^-CN^−^ Concentrations (mg/L) ^†^
	1st	2nd	
0.1 M CN as KCN	Deionized water	0.001 M Ag_2_SO_4_	0.054 ± 0.001
		0.002 M Ag_2_SO_4_	0.044 ± 0.003
		0.003 M Ag_2_SO_4_	0.014 ± 0.002
		0.004 M Ag_2_SO_4_	0.007 ± 0.002

^†^ MB^+^CN^−^ concentration, equivalent to the interfering concentration of anions, was calculated by using the absorbance of each MB^+^CN^−^ solution and interpolating the values into an SDS standard curve.

**Table 5:** Effect of sulfuric acid pretreatment on COO^−^ interferences in MIBK-DCE extraction.

Anions and Treatment Concentrations (M)	pK_a_ *	MB^+^A^−^ (mg/L) ^†^
Acetate, 1.0	a_1_ = 4.76	−0.004 ± 0.000
Tartrate, 0.5	a_1_ = 3.04, a_2_ = 4.37	0.003 ± 0.003
Citrate, 0.5	a_1_ = 3.13, a_2_ = 4.76, a_3_ = 6.40	0.020 ± 0.001
Benzoate, 0.02	a_1_ = 4.20	0.026 ± 0.000
Benzoate, 0.05		0.059 ± 0.002
Salicylate, 0.0002	a_1_ = 2.98	0.044 ± 0.003
Salicylate, 0.002		0.122 ± 0.001
Biphthalate, 0.002	a_1_ = 2.95, a_2_ = 5.41	0.553 ± 0.001
Biphthalate, 0.02		1.434 ± 0.034

* pK_a_ = −log (K_a_); K_a_ is acid dissociation constant [32]. ^†^ MB^+^A^−^ concentration, which is equivalent to interfering concentration of anions, was calculated by determining the absorbance of each MB^+^A^−^ solution and interpolating it into the SDS standard curve.

**Table 6:** Effects of washing with carbonate–bicarbonate buffer solution on the interferences by biphthalate and salicylate.

Anions	Concentrations (M)	1st Washing and pH		2nd Washing	MB^+^A^−^ Interfering Concentration ^†^ (mg/L)
Biphthalate	0.005	D-water	6.1	Deionized water	1.013 ± 0.015
		Carbonate–bicarbonate buffer solution	9.2	Deionized water	0.030 ± 0.002
Salicylate	0.01	D-water	6.1	Deionized water	0.496 ± 0.002
		Carbonate–bicarbonate buffer solution	9.2	Deionized water	0.395 ± 0.002

^†^ MB^+^A^−^ concentration, equivalent to the interfering concentration of anions, was calculated by using the absorbance of each MB^+^A^−^ solution and interpolating the values into an SDS standard curve.

**Table 7:** Effects of first and second washing reagent on the interferences by salicylate.

Anion	Washing Reagents		MB^+^A^−^ Interfering Concentration ^†^ (mg/L)
	1st Washing	2nd Washing	
Salicylate (0.01 M)	Carbonate–bicarbonate buffer solution at pH 9.2	Deionized water	0.294 ± 0.013
		0.1% Ag_2_SO_4_	0.317 ± 0.040
		0.1% Ag_2_SO_4_ + 0.005 M Al^3+^	0.113 ± 0.004
		0.1% Ag_2_SO_4_ + 0.005 M Mn^2+^	0.283 ± 0.017
		0.1% Ag_2_SO_4_ + 0.005 M Zn^2+^	0.303 ± 0.005

^†^ MB^+^A^−^ concentration, equivalent to the interfering concentration of anions, was calculated by using the absorbance of each MB^+^A^−^ solution and interpolating the values into a SDS standard curve.

**Table 8:** QA/QC data by MIBK-DCE method and chloroform method.

QA/QC Criteria		MIBK-DCE Method	Chloroform Method
SDS Standard Curve	Slope	0.6396	0.5252
	r^2^	0.9999	0.9995
Detection Limit	MDL ^1^	0.0001	0.0041
	LOQ ^2^	0.0005	0.0137
Accuracy (%, recovery)	Low Concentration ^3^	104.7	96.5
	Medium Concentration ^4^	99.0	83.3
Precision (%, RSD ^5^)	Low Concentration	0.2	7.1
	Medium Concentration	0.1	13.9

^1^ MDL = method detection limit. ^2^ LOQ = limit of quantitation. ^3^ Low concentration: 8–10 samples having 0.02 mg SDS/L were used for determination of LOQ. ^4^ Medium concentration: 4–7 samples having 0.2 mg SDS/L were used for determination of accuracy and precision of the method. ^5^ RSD: relative standard deviation.

**Table 9:** Recovery (%) of SDS by MIBK-DCE method for different environmental water samples.

Type of Water Samples	Locations	Spiked SDS Conc. (mg/L)	Measured SDS Conc. (mg/L)	Recovery (%)
Groundwater	Seomyeon, Chuncheon	0.000	0.012 ± 0.004	-
		0.3~0.9	-	97.5–98.2
Stream water	Namdaecheon, Yangyang	0.0	0.004 ± 0.005	-
		0.3~0.9	-	97.8–98.4
	Gongjicheon, Chuncheon	0.0	0.009 ± 0.000	-
		0.2~1.2	-	99.8–101.8
Seawater	Namhyangjin, Gangreung	0.000	0.008 ± 0.000	-
		0.2~1.2	-	100.1–103.2
	Sacheon, Gangreung	0.000	0.003 ± 0.001	-
		0.1~0.5	-	97.2–100.7
Influent of wastewater treatment plant	Gangreung	0.000	0.250 ± 0.001	-
		0.3~0.9	0.537 ± 0.005	93.1–95.7
Effluent of wastewater treatment plant	Gangreung	0.000	0.021 ± 0.001	-
		0.3~0.9	0.317 ± 0.002	98.2–98.6

**Table 10:** Comparison of scale of operation between MIBK-DCE method and chloroform method in SDS analysis (*n* = 12).

Process	MIBK-DCE Method	Chloroform Method	Remarks ^1^
Total analytical time (min)	90	270	2/3 reduction
Solvent requirement (mL)	600	1390	1/2 reduction
Apparatus requirement (ea)	12	36	2/3 reduction

^1^ Reduction in analytical time as compared to Korea standard method (MOE, 2017).

## Data Availability

Not applicable.

## Supplementary Materials

The following supporting information can be downloaded at: https://www.mdpi.com/article/10.3390/toxics10040162/s1, Table S1: Wastewater samples and sampling locations used for the sensitivity analysis of the MIBK-DCE method.
