# Supplementary material for: A Simplified Method for Anionic Surfactant Analysis in Water Using a New Solvent
Source: Toxics. 2022 Mar 29;10(4):162. doi: 10.3390/toxics10040162 (PMC9025779; doi:10.3390/toxics10040162)
Supplement: Supplementary file 1 [file toxics-10-00162-s001.zip › toxics-1659032-supplementary.pdf]

toxics

MDPI

Supplementary Materials: A Simplified Method for Anionic Surfactant Analysis in Water Using a New Solvent

Jung-Hwan Yoon, Yong-Geon Shin, Mary Beth Kirkham, Seok-Soon Jeong, Jong-Geon Lee, Hyuck-Soo Kim and Jae E. Yang

Table S1. Wastewater samples and sampling locations used for the sensitivity analysis of the MIBK-DCE method.

No	Water Sample	Sampling Location
	Industrial wastewater	
1	Car wash wastewater	Wonju-si
2	Car wash wastewater	Wonju-si
3	Car wash wastewater	Wonju-si
4	Car wash wastewater	Wonju-si
5	Car wash wastewater	Wonju-si
6	Car wash wastewater	Wonju-si
7	Car wash wastewater	Wonju-si
8	Car wash wastewater	Wonju-si
9	Car wash wastewater	Chuncheon-si
10	Car wash wastewater	Chuncheon-si
11	Car wash wastewater	Gangneung-si
12	Car wash wastewater	Taebaek-si
13	Laundry wastewater	Jeongseon-gun
14	Car wash wastewater	Jeongseon-gun
15	Car wash wastewater	Jeongseon-gun
16	Car wash wastewater	Jeongseon-gun
17	Car wash wastewater	Jeongseon-gun
	Domestic sewage water	
1	Sewage treatment plant influent	Sokcho-si
2	Sewage treatment plant effluent	
3	Village scale sewage plant influent	Samok-ri, Yeongwol-eup, Yeongwol-gun
4	Village scale sewage plant effluent	
5	Village scale sewage plant influent	Geoun-ri, Yeongwol-eup, Yeongwol-gun
6	Village scale sewage plant effluent	
7	Village scale sewage plant influent	Sillim-myeon, Wonju-si
8	Village scale sewage plant effluent	
9	Chuncheon sewage treatment plant effluent	Chuncheon-si
10	Gangchon sewage treatment plant effluent	Chuncheon-si
11	Seomyeon sewage treatment plant effluent	Chuncheon-si
12	Sinbuk sewage treatment plant effluent	Chuncheon-si
13	Village scale sewage plant influent	Guam-ri, Yanggu-gun
14	Village scale sewage plant effluent	
15	Village scale sewage plant influent	Sokcho-ri, Hongcheon-gun
16	Village scale sewage plant effluent	
17	Village scale sewage plant influent	Yuchi-ri, Hongcheon-gun
18	Village scale sewage plant effluent	
19	Environmental fundamental facility influent	Hongcheon-gun

20	Environmental fundamental facility effluent	
21	Village scale sewage plant influent	Hakjo-ri, Yanggu-gun
22	Village scale sewage plant effluent	
23	Village scale sewage plant influent	Andae-ri, Yanggu-gun
24	Village scale sewage plant effluent	
